# A process for developing multisectoral strategies for zoonoses: the case of leptospirosis in Fiji

**DOI:** 10.1186/s12889-017-4673-1

**Published:** 2017-08-22

**Authors:** Simon A. Reid, Anna Rodney, Mike Kama, Peter S. Hill

**Affiliations:** 1 The University of Queensland, School of Public Health, Herston, QLD 4006 Australia; 2 Fiji Centre for Communicable Disease Control, Ministry of Health and Medical Services, Suva, Fiji

**Keywords:** Leptospirosis, One Health, Zoonotic diseases, Governance, Collaboration, Multi-sectoral

## Abstract

**Background:**

Zoonotic diseases such as leptospirosis occur as a result of the often complex interactions that exist at the human-animal-environment interface. The most obvious consequence of this complexity is the need for the health sector to partner with institutions in other sectors of society such as agriculture, labour and local government. This multisectoral engagement is complicated by the different agendas and cultures of the various institutions and their ability to “see” their role and ant benefits in a collaborative response.

**Methods:**

The research used a realist review methodology combined with systems thinking frameworks to determine the optimal strategy and governance for the prevention and control of leptospirosis in Fiji. The process included facilitated workshops with multiple stakeholders to determine the needs, issues and potential interventions that was guided by a synthesis of locally available data and information on the impact of leptospirosis. This process was informed by interviews with bureaucrats from different government ministries.

**Results:**

Stakeholders concurred that leptospirosis generally only received wide-spread attention in outbreaks, when there is media coverage of deaths or a large number of hospitalisations. In general, all ministries expressed support for a multisectoral strategy but saw the Ministry of Health and Medical Services as the lead agency with overall responsibility. The final consultation workshop yielded a clearly articulated goal to reduce the case fatality rate attributable to leptospirosis by 50% by 2020 and 4 overarching strategies: 1) improved clinical management of leptospirosis, 2) improved surveillance for leptospirosis, 3) enhanced communication to minimise risk and improve health seeking behaviours, and 4) strengthening coordination and governance structures.

**Conclusion:**

Human mortality and morbidity remained the primary drive for government action, defining leptospirosis as a human health problem. The process of deliberative consultation, and the engagement of multidisciplinary partners has provided a platform for collaborative policy development, and a consensus for a National Action Plan from which further negotiated collaboration will be possible.

**Electronic supplementary material:**

The online version of this article (doi:10.1186/s12889-017-4673-1) contains supplementary material, which is available to authorized users.

## Background

Leptospirosis is a globally important zoonotic disease, caused by infection with bacteria of the genus *Leptospira*, that occurs mainly in tropical or sub-tropical countries in both urban and rural settings [1]. Rodents (rats and mice) and domestic mammals, such as cattle, pigs and possibly dogs are the major reservoir hosts [2]. Fiji has one of the highest leptospirosis burdens in the region with an average annual incidence of 45.6 Immunoglobulin M ELISA-positive (IgM ELISA-positive) cases per 100,000 and approximately 31 deaths from 2012 to 2014 (Table 1) based on data from the Ministry of Health and Medical Services (MHMS) (MHMS, unpublished).

**Table 1:** The number of IgM ELISA-positive cases of leptospirosis and attributed deaths in Fiji, 2008–2014 (MHMS, unpublished)

	2008^a^	2009	2010^a^	2011	2012	2013	2014
Cases	24	152	5	107	563	453	127
Deaths	21	31	15	51	38	39	16

^a^Case data incomplete

Leptospirosis shows strong seasonality in parts of Fiji and outbreaks tend to be associated with flooding or heavy rainfall. For example, in 2012, more than half of the country’s cases were reported from the Western Division following two flooding events [3]. The disease is over-represented amongst indigenous (iTaukei) Fijian males aged between 15 and 45, thought to be due to higher exposure due to occupational and recreational behaviours [4]. A recent serological study demonstrated that this demographic had a higher prevalence of antibodies to *Leptospira* that was associated with working outdoors, residing in rural areas and the presence of pigs and cattle [5]. Infection spreads indirectly between animals and humans through contact with water or mud contaminated by infected urine from rodents or domestic animals including cattle, dogs, horses and pigs [6, 7]. For Fiji’s farming sector—traditional and modern—the economic cost to production is compounded by the health costs for livestock and agricultural workers. There has been no systematic evaluation of the burden of disease attributed to leptospirosis and the associated economic impacts, which complicates efforts to set national-level priorities.

Leptospirosis is a preventable disease and control measures must include education and targeted awareness campaigns for specific risk groups [8], improvements in sanitation and hygiene, environmental modification to reduce standing water, as well as rodent control [9, 10], but responsibility for the animal reservoirs and possible solutions lies with institutions and individuals that are not in the health sector. As such, a national strategy which encompasses interventions to meet the needs of the different stakeholders, especially those associated with the animal reservoirs, is an important tool for advocacy for institutional action.

In 2011, the MHMS began a process to develop a multisectoral national strategy by convening two consultation workshops. These meetings aimed to create a set of mutual goals and objectives for implementation of a national strategy for the control of leptospirosis by identified organizations and key government departments. The meetings also sought to determine how to establish a collaborative network and what sort of coordinating body should drive the process. It became apparent, however, that a lack of information about leptospirosis was an issue which was limiting stakeholders’ ability to make significant commitments to the national strategy. Subsequently, in 2013 the MHMS, in conjunction with the World Health Organization (WHO), coordinated a meeting of international experts which sought to review the epidemiology of leptospirosis in Fiji and recommend priorities for the control of endemic and epidemic disease.

In 2014, researchers from the University of Queensland collaborated with the MHMS in research intended to inform and catalyse the preparation of the national strategy for the prevention and control of leptospirosis, culminating in a final collaborative workshop to identify specific interventions that would be pursued by the government of Fiji up to 2020. This paper examines the processes leading to the final national strategy, exploring the role played by a realist review undertaken in collaboration with the Fiji Centre for Communicable Disease Control (FCCDC) in the development of the plan and its governance.

## Methods

### Data sources and analysis

Data on individual laboratory-confirmed cases of leptospirosis were obtained from the FCCDC for the period from 2008 to 2014 (unpublished observations). The FCCDC tests each serum from each suspected case of leptospirosis with the Panbio® Leptospira IgM ELISA (Alere, Waltham, MA). Additional data included the age, gender, ethnicity, the dates of sample collection, receival and reporting and the IgM ELISA result as a dichotomous outcome.

All reported deaths due to leptospirosis were obtained from the Health Information Unit, MHMS (unpublished observations) using the underlying cause of death of leptospirosis icterohemorrhagica (A27.0), other forms of leptospirosis (A27.8) and leptospirosis, unspecified (A27.9) according to the International Classification of Diseases, Tenth Revision (ICD-10) [11]. Demographic data for Fiji were obtained from the Fiji Bureau of Statistics [12].

A descriptive analysis of laboratory-confirmed cases and reported deaths was performed to determine major demographic trends in the data. In addition, a crude case fatality rate calculated using the laboratory-confirmed “cases” and reported deaths as total “fatalities” and further descriptive analysis performed.

Data on leptospiral serovars in reservoir animal hosts in Fiji was available from an unpublished research report [13].

### Realist review methodology

The research used a realist review methodology [14, 15], informed by the systems thinking approach described by D De Savigny and T Adam [16]. The process followed the five steps outlined in the realist review approach:

- Step 1: Clarify the scope and define the intentions of the review, and formulate the explanatory theories to be explored in the review
- Step 2: Search for evidence that might reasonably test these theoretical constructs
- Step 3: Appraise the available primary research and extract the data relevant to the review
- Step 4: Synthesise this evidence and draw conclusions
- Step 5: Disseminate, implement and evaluate these conclusions [11, 12].

#### Step 1: Clarification and theorising

The research question for the review was determined by the MHMS’s expressed policy needs, arising out of a series of seasonal leptospirosis outbreaks documented since 2008, with significant mortality: what is the optimal strategy and governance for the prevention and control of leptospirosis in Fiji? The scope of the review was to address both strategy--dealing with both acute outbreak and chronic endemic infection—and governance—given the complex multi-sectoral responses needed to manage leptospirosis.

From the targeted literature review, synthesis of available data on IgM ELISA-positive cases, reported deaths due to leptospirosis, data on serovars in animal reservoirs, and discussions with staff of the FCCDC we theorised that:

1. Leptospirosis has significant impacts both on human mortality and morbidity but also on economic drivers (sugar, dairy, traditional agriculture and potentially tourism);
2. That a limited number of government agencies are directly involved, but that effective coordination in this multi-sectoral problem should probably be at a higher level than the MHMS; and
3. That structured presentation of evidence, and a greater range of metrics addressing sectoral interests would be the necessary catalyst for national policy change.

#### Step 2: Search for evidence

The search for evidence that would inform potential options in strategy and test the formulated theories was conducted in three parts: the first was an analysis of the available epidemiological and clinical data on leptospirosis in Fiji, from information systems in health and agriculture; the second was a qualitative research process interviewing key informants from government and industry agencies with an interest in leptospirosis; the third was the calculation of the Years of Life Lost due to leptospirosis, and an estimate of the willingness to pay to avoid this loss.

The analysis of available data was undertaken in collaboration with the MHMS and the Ministry of Agriculture, the WHO and University of Queensland researchers. The findings are outlined in the introductory background section of this paper.

The qualitative research was undertaken in 2014, with 18 key semi-structured key informant interviews representatives of government agencies and relevant industry organisations (see Additional file 1: Appendix 1 for interview guide). The informants selected were either nominated by the ministry or organisation; identified from the output documents of the 2011 and 2013 workshops; or snow-balling based on the knowledge and experience of local professionals in the animal and human health fields. They included senior bureaucrats in the following ministries: Health (7), Agriculture (3), Information and Communication (1), Labour (1), iTaukei Affairs (1), and the Department of Environment within the Ministry of Local Government (1). Interviews were also conducted with private industry groups including the Fiji Cooperative Dairy Company Ltd. (FCDCL) and the Sugar Cane Growers Council (SCGC), a sugar cane-farmer and a former senior government veterinarian currently representing several non-governmental organizations (NGOs) and committees. Interviews used a common interview question guide, adapted to the specific context of the informant, and were conducted at their work location, recorded with consent for transcription.

All interviews were transcribed and de-identified at the point of analysis, and a code substituted. The code record was kept under a password protected file by the researcher undertaking the analyses (Anna Rodney). The transcripts underwent thematic analysis using NVivo 11 Pro (QSR International, Melbourne, Australia). A priori themes were developed consistent with the question guide with emergent themes added as they were identified in the analysis. The themes were corroborated by a second analyst (Peter Hill).

The thematic analysis formed the basis from which the theories we had articulated could be tested, with distinctive patterns and responses emerging from the different sectors and agencies, and differing perspectives evident from different disciplines and organizations. Five themes were evident in the analysis:

1. Perceptions of the burden and impact of leptospirosis and available evidence
2. Decision-making and governance processes for infectious disease outbreaks
3. Multi-sectoral collaboration models for health
4. Perceptions of key stakeholders for leptospirosis control
5. Leptospirosis control and interventions.

The essential findings suggested a broad recognition of the mortality associated with acute outbreaks of leptospirosis, but poorly defined understanding of the impact across sectors, and limited and uneven evidence for this. Multi-sectoral collaboration was seen to be effective in acute outbreaks but required higher level governance to sustain collaboration during the endemic phases. Despite the awareness of a need for a collaborative approach, health actors continued to be seen as the prime stakeholders, with control largely targeted at clinical interventions. These perspectives were demonstrably challenged through the review process.

We then collectively brainstormed potential priorities and interventions and then conceptualized the effects (step 3) of these on the burden of leptospirosis. The final phase was to adapt and redesign (step 4) the interventions to optimize synergies and other positive effects while avoiding or minimizing any potentially major negative effects.

#### Step 3: Appraise the research and extract the data relevant to the review

A final stakeholder synthesis workshop was held in August 2015, which drew together the outputs of the previous activities and the outputs of a number of research projects to formulate this action plan. This final consultation focused on the development of an action plan to articulate with the National Strategic Plan (2016–2020) of the MHMS [17]. At this workshop, discussed in detail in our findings, we convened stakeholders representing each component of the leptospirosis “system”, identified by their involvement in the evolution of the problem (i.e. agricultural sectors) or their role in the management of a response (i.e. government agencies (health, agriculture, environment) and agricultural sectors). The findings of the available epidemiological, clinical and economic data were presented synthesising recent research information—including the data from the key informant interviews and economic data--with existing knowledge to identify and priorities specific actions that would have the greatest impact on the fatality rate of leptospirosis.

#### Step 4: Synthesize evidence and draw conclusions

From discussions following the presentation of the data, four common and inter-related strategic themes emerged that would shape planning and policy:

- Strengthened diagnosis, clinical management and case investigation
- Enhanced surveillance and information systems
- Health promotion/risk communication to reduce risky behaviours and to promote early presentation of cases
- Multi-sectoral collaboration

Having established these themes, participants were led through a process that again reviewed new and existing knowledge and created a comprehensive list of actions, and clarified the governance structures needed for implementation. Discussion groups were formed for each of the four themes, facilitated by a local professional with expertise in each particular theme. From the comprehensive list, selected actions relevant to each theme were then ranked using 4 criteria, (cost, impact, feasibility and capacity), scoring them from 1 (immediately implementable) to 3 (implementation constrained) (Table 2). Findings of the discussion groups were corroborated by participants from each sector represented, and in a plenary presentation.

**Table 2:** Ranking criteria and scores used to select interventions for Fiji’s leptospirosis strategy

Cost 1. Within current MHMS resources/budget 2. With likely additional MHMS resources/budget 3. Needs external (outside MHMS) resources	Impact by 2020 1. Will directly reduce mortality 2. Will indirectly but predictably reduce mortality 3. May reduce mortality
Feasibility 1. We are able to do this now 2. There are minor obstacles to achieve this 3. Significant obstacles to be overcome	Human Resources/Capacity 1. We have the resources and capacity now 2. Current staff need additional training to achieve this 3. New capacity needs to be introduced

This process synthesized the best current evidence on the local epidemiology of the disease and most effective interventions, with actions ranked with the lowest combined scores (less than 9) deemed priority interventions that participants felt that could feasibly be achieved in 5 years or less. The reports of each of the groups, with their rankings of priority based on the four parameters (cost, impact, feasibility and capacity) were used as the basis of development of the National Action Plan for Leptospirosis, incorporating the revised theoretical framing emerging from the workshop.

#### Step 5: Disseminate, implement and evaluate

The National Action Plan for Leptospirosis was presented to the Permanent Secretary for Health before being submitted to the Planning and Policy Development Division of the MHMS for review and endorsement.

## Results and Discussion

The findings presented here essentially combine steps 3 and 4 in the realist review process described, integrating a summary of the evidence presented to the national stakeholder workshop in August 2015, and its synthesis into four strategic themes for the National Action Plan, and the prioritization of interventions within each of those themes. One theme that was not strongly represented in this study was the management of environmental risk factors for leptospirosis particularly, water-shed management, appropriate location of livestock and agricultural practices. These areas will be pursued separately as additional epidemiological data become available.

Data on the incidence of leptospirosis used in preliminary activities is constrained by the difficulty in clinical and laboratory diagnosis, in particular the use of the IgM ELISA as a “confirmatory” test, which is not in accordance with the recommendation of the Leptospirosis Burden Epidemiology Reference Group (LERG) [18]. However, improving clinical and laboratory diagnosis was identified as a high priority and will be addressed through the formulation of new clinical management guidelines that will incorporate standardised case definitions based on the LERG.

### Perceived burden and impact

Stakeholders concurred that leptospirosis generally only receives wide-spread attention in outbreaks, when there is media coverage of deaths or a large number of hospitalisations. In terms of affected and at-risk populations, health stakeholders agreed the disease was over-represented amongst iTaukei males aged 15–29 and despite acknowledging that there was a paucity of research on routes of infection, this was anecdotally reported to be associated with high-risk employment activities, such as cane harvesting, domestic gardening and to a lesser extent livestock husbandry, and recreational activities, particularly swimming. This is in agreement with the findings of a review by Ram and Collings [4] who concluded high prevalence of infection in young (20–29) indigenous Fijian males, compared to the low rates in Indo-Fijians, strongly suggested a link with the place and nature of work and possibly to specific risk factors such as time spent in water (e.g. fishing) or walking barefoot etc.

Risk was reported to be highest after flooding or around the rainy season and the cane harvesting season, which is in keeping with historical reports of increased cases numbers during the rainy season [19]. It was also observed that after the 2012 floods there were initially clusters of disease in rural areas which were followed by cases of disease in urban areas, a pattern which was thought to be due to the movement of rats into the peri-urban areas in search of food and shelter. Whilst there are no data on the dynamics of rodent populations in 2012 the perceived role that rodents play as reservoirs for human disease is partially supported by the scant published data. For example, a historical review of human cases in the 1950’s identified that the majority were attributed to serovars *icterohaemorrhagica*, *australis* and *canicola* [19] and a recent survey also identified that dogs and humans in one village had antibodies to serovars *canicola*, *australis* and *copenhageni* [13]. These serovars have largely been associated with rodent reservoir hosts [7].

Despite the perception that there are associated economic and social costs across multiple sectors [20], all stakeholders viewed leptospirosis primarily as a problem purely associated with the health impacts of human morbidity and mortality.

It is possible that there were other stakeholders omitted from the process that held a different perspective to those identified by the respondents, in particular, the communities at risk of leptospirosis. The research team has undertaken qualitative research to explore the perspectives and needs of cases and the families of cases of leptospirosis. The results of this study will be published elsewhere and were not available at the time of the consultation workshops. The results of that study showed that the majority of cases could not identify factors associated with their infection and, of those that could, only one was associated with animal contact (Reid et al., unpublished). The needs expressed by this group were largely for improved health service provision and information on methods to modify agricultural practices to reduce risk. Both these areas were identified during the process described in this paper.

This research selected diverse representation from within government agencies, health services and relevant industry. The scope from within government was extensive with interviews and workshop participants drawn from 6 ministries and the three largest agricultural sector organisations (sugar, dairy and crops and livestock (non-sugar agriculture). The authors are not aware of additional groups that have a significant stake in the issue of leptospirosis in Fiji. In addition, the consistency of responses during the interviews and lack of dissent during the final workshop suggest that an expanded stakeholder group may not have provided new perspectives.

### Evidence for decision making and the current flow of information on leptospirosis

Stakeholders from within the health system acknowledged that the data and evidence on leptospirosis collected by the National Notifiable Disease Surveillance System (NNDSS) is poor and that leptospirosis is likely to be significantly under-reported for several reasons. Firstly, leptospirosis is difficult to differentiate clinically from the more common febrile diseases such as dengue and typhoid, with clinical informants indicating that doctors generally have less knowledge and awareness of leptospirosis than the other two diseases. It was also reported that doctors needed further training on accurate coding of leptospirosis and other infectious diseases according to the International Classification of Diseases (ICD-10). Internal reporting of confirmed cases from the divisional laboratories was also described to be sporadic at best, and data from private clinics, hospitals or laboratories is not integrated into the NNDSS. For the data that the NNDSS does routinely collect, it aggregates the numbers of cases per facility but does not have the capacity to collect demographic information on individuals—many of whom are highly mobile—or their health seeking behaviours. While it was reported the National Taskforce for the Control of Outbreak-Prone Diseases (NTCOPD) is discussing the introduction of a new format for recording such information, this had not yet been implemented. The MHMS did have 2 years of research data showing the areas/villages from which people who died of leptospirosis originated, however without routine collection of such information, high risk populations cannot be effectively defined and targeted with preventative messages and interventions. The MHMS informants unanimously agreed they needed further funding and resources to be able to combat the ‘three plagues’: dengue, leptospirosis and typhoid. It was also felt that, with increased human resources, it would be ideal to create a dedicated team to manage leptospirosis that could collate and synthesise the results of several clinical and epidemiological research projects that are being undertaken by a range of partners.

### Priorities for 5-year the strategy

The final consultation workshop yielded a clearly articulated goal and 4 overarching strategies, consistent with the focus on epidemic mortality, and provided an opportunity to combine theoretical constructs and the evidence presented for policy. The review of evidence and the outcomes of the workshop confirmed our first theoretical construct on the perceived significant impact of leptospirosis. The second proposal, that coordination in this multi-sectoral problem should be at a higher level than the MHMS recognised the leadership of MHMS in acute outbreaks of leptospirosis, and proposed a collaborative governance structure lead by the MHMS that would address broader structural reforms. Support for the third theoretical construct was ambiguous: while the collation and presentation of the available evidence clearly contributed to endorsement of national policy change, there was little appetite for a commitment to a greater range of metrics addressing sectoral interests.

The goal “to reduce the case fatality rate attributable to leptospirosis by 50% by 2020” was considered by the participants as the most effective target for reducing the burden in the relatively short 5-year timeframe. The participants did not feel that there was sufficient knowledge regarding major risk factors, including individual’s behaviours and practices, to justify pursuing the broader goal of reducing the number of cases (incidence). This was considered to be a longer term goal.

To achieve the reduction in case fatality rate, the following major strategies will be pursued:

1. Improved clinical management of leptospirosis
2. Improved surveillance for leptospirosis
3. Enhanced communication to minimise risk and improve health seeking behaviours
4. Strengthening coordination and governance structures.

#### Strategy 1: Improved clinical management of leptospirosis

This strategy focused on creating systems and guidelines to improve the early detection and consistency of case management (diagnosis, treatment and referral). The identified interventions included the development of triaging system based on syndromic case detection, development of guidelines and protocols for use of rapid diagnostic tests (RDTs) in clinical settings, and promotion of simplified clinical guidelines applicable to each level of the health system.

#### Strategy 2: Improved surveillance for leptospirosis

The two contexts considered in this strategy were cases associated with “outbreaks” or disasters and endemic/sporadic transmission. Active surveillance, including active case finding and investigation (outreach), was considered necessary in disaster settings to ensure early detection of suspected cases. The quality of data was identified as a significant impediment to good planning, so the MHMS will work to standardise the process of notification and case investigation, including standardised data collection tools and training of environmental health officers who perform investigations. Revision of the diagnostic capacity at the Divisional hospitals (IgM ELISA or RDTs) and the Fiji Centre for Communicable Disease Control (FCCDC) (IgM ELISA and polymerase chain reaction) were considered necessary to increase the speed of laboratory testing.

#### Strategy 3: Activities to minimise risk and improve health seeking behaviours

This strategy was largely directed towards improving knowledge of leptospirosis (and febrile illness more generally) in order to reduce risky behaviours and more importantly, increase early presentations at health facilities. This would be achieved through a collaborative strategic health communication plan that will be designed to improve the coverage, capabilities and reach of risk communication between sectors during the endemic periods between outbreaks, as well as in during epidemics and related disasters. This would involve the development of new cross-sectoral partnerships that would include stakeholders such as the new Fiji Crop and Livestock Council. A significant effort to build capacity for community/village health workers to provide communication and behaviour change programs was considered essential to the success of this strategy.

#### Strategy 4: Strengthening coordination and governance structures

**Fig. 1:**
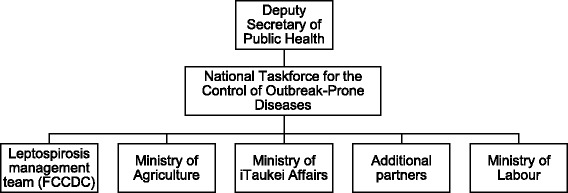
Governance structure for the management of the Fiji National Action Plan for Leptospirosis

The strongest proponents of a new governance structure were the senior government staff from the key ministries. This was seen as a critical feature of the plan, and would need to have a specific mandate. Within the MHMS a leptospirosis management team would be based at the FCCDC with nodes in each Division responsible to National Taskforce for Control of Outbreak Prone Diseases and Deputy Secretary of Public Health (see Fig. 1). In addition, to ensure cross-sectoral and cross-institutional collaboration, memoranda of understanding with other ministry stakeholders (Ministry of Agriculture etc) would be drafted to enable engagement (for communication) and rapid response in the event of outbreaks/disasters.

## Conclusions

The combination of realist review with a national planning process enabled the building of multi-sectoral consensus and a commitment to a National Action Plan for Leptospirosis, even though the original hypotheses required revision at the conclusion of the process. Stakeholders conceded the probable economic impact in their sectors, but epidemic mortality continued to shape their perceptions of the policy significance of leptospirosis. The economic analyses demonstrated significant costs, but did not tangibly influence perceptions of priorities or governance. While there is a broader governance constituency for leptospirosis than previously imagined, human mortality and morbidity remain the primary drive for government action, defining leptospirosis as a human health problem. The ad hoc default to MHMS leadership in epidemic contexts, and the responsive cooperation of other agencies in the acute outbreak, was seen to provide continuity into the management of the endemic intervals between epidemics. While coordination and communication have been problematic during the endemic phase, the process of deliberative consultation, and the engagement of multidisciplinary partners has provided a platform for collaborative policy development, and a consensus for a National Action Plan from which further negotiated collaboration will be possible.

## Additional file

Additional file 1: Reid et al. Leptospirosis in Fiji Supplementary appendix 1. Interview guide used as part for the process to develop a national strategy for leptospirosis in Fiji. (DOCX 14 kb)

## Abbreviations

ELISA: Enzyme-linked immunosorbent assay
FCCDC: Fiji Centre for Communicable Disease Control
FCDCL: Fiji Cooperative Dairy Company Ltd.
ICD-10: International Classification of Diseases, Tenth Revision
IgM: Immunoglobulin M
LERG: Leptospirosis Burden Epidemiology Reference Group
MHMS: Ministry of Health and Medical Services
NGOs: Non-governmental organizations
NNDSS: National Notifiable Disease Surveillance System
NTCOPD: National Taskforce for the Control of Outbreak-Prone Diseases
RDTs: Rapid diagnostic tests
SCGC: Sugar Cane Growers Council
WHO: World Health Organization
