# Supplementary material for: A process for developing multisectoral strategies for zoonoses: the case of leptospirosis in Fiji
Source: BMC Public Health. 2017 Aug 22;17:671. doi: 10.1186/s12889-017-4673-1 (PMC5567884; doi:10.1186/s12889-017-4673-1)
Supplement: Additional file 1: — Reid et al. Leptospirosis in Fiji Supplementary appendix 1. Interview guide used as part for the process to develop a national strategy for leptospirosis in Fiji. (DOCX 14 kb) [file 12889_2017_4673_MOESM1_ESM.docx]

**Appendix 1.** Interview guide used as part for the process to develop a national strategy for leptospirosis in Fiji

1. **Introduction**

- Respondent’s position and background

1. **Perceptions of leptospirosis impacts**

- What do they perceive to be the main impacts of leptospirosis (size, effects, impacted groups, economy etc)?
- What are the main impacts for their sector/area of control?
- What is the evidence base for these views? What data/information is available?
- What are the main impacts for other sectors/areas?
- What information/evidence do you require when making decisions associated with leptospirosis?

1. **Key stakeholders**

- Who do they see as the (3) key stakeholders involved in leptospirosis control?
- What relationships do they have with these stakeholders?

1. **National strategic plan (NSP)**

- Were they/their organisation involved in the stakeholder meeting in October 2011?
- What do they feel were the main outcomes of the meeting?
- What has happened since? Have there been any progress/changes in linkages with other stakeholders?
- What do they feel are the key interventions?
- Do they feel the current climate is acceptable for finalisation and implementation of the NSP? What are barriers/enablers?
- Who do they see as their organisations commitments and contributions to the plan?
- What sort of expectations do they have in terms of resourcing the NSP?
- What steps should be taken next?
- Who should have carriage of the NSP?
- Who should sit on a core intersectoral working group for the NSP?
- Who does leptospirosis really matter for?

1. **Collaboration/communication**

- What are the barriers to intersectoral communication and collaboration?
- From your opinion what are the opportunities for establishing intersectoral collaboration? How could it be done?
- Who should be the lead/driver of the process?
